# Left Ventricular Mechanics in Multivessel Coronary Artery Disease with Versus Without Chronic Total Occlusion

**DOI:** 10.3390/jcdd13090449

**Published:** 2026-09-09

**Authors:** Stela Madunic, Tina Volarevic, Mihajlo Kovacic, Antonia Melada, Dino Miric, Josip Andelo Borovac

**Affiliations:** 1 School of Medicine, University of Split, 21000 Split, Croatia; stela.madunic@gmail.com (S.M.); tina.volarevic@kb-merkur.hr (T.V.); dino.miric@kbsplit.hr (D.M.); 2 Department of Interventional Cardiology, County Hospital Cakovec, 40000 Cakovec, Croatia; voditelj.intervencijska.kardiologija@bolnica-cakovec.hr; 3 Division of Clinical Cardiology, Department of Cardiovascular Diseases, University Hospital of Split (KBC Split), 21000 Split, Croatia; antonia.melada@kbsplit.hr; 4 Division of Ischemic Heart Disease, Department of Cardiovascular Diseases, University Hospital of Split (KBC Split), 21000 Split, Croatia

**Keywords:** chronic total occlusion, coronary artery disease, global longitudinal strain, myocardial work, left ventricular function, echocardiography

## Abstract

Chronic total coronary occlusion (CTO) occurs within advanced coronary artery disease (CAD), but its association with left ventricular (LV) deformation and pressure–strain-loop-derived myocardial work beyond global LV ejection fraction (LVEF) is uncertain. We performed a retrospective cross-sectional analysis of 148 unique patients with multivessel or left-main CAD, including 70 with and 78 without CTO. Absolute global longitudinal strain (GLS) was the principal endpoint; global work index (GWI), constructive work (GCW), wasted work (GWW), and work efficiency (GWE) were secondary endpoints. Sequential CTO coefficients were estimated using HC3 heteroscedasticity-consistent linear models in a pool of patients with complete global mechanics and LVEF data. A history of myocardial infarction was more frequent with CTO (42.9% vs. 23.1%), with lower LVEF and larger LV volumes. In crude distributional comparisons among 136 patients with global mechanics data, median GLS was 14.0% versus 15.5% (*p* = 0.021), while median GCW was 1723 versus 1997 mmHg% (*p* = 0.026). In the multivariable regression analysis, the CTO-minus-non-CTO GLS coefficient was −1.40 percentage points (95% CI, −2.80 to 0.00) unadjusted, −1.10 (95% CI, −2.58 to 0.38) after clinical adjustment, and −0.16 (95% CI, −1.24 to 0.92) after adding LVEF. CTO status marked a modest adverse global LV mechanical phenotype, but clinically adjusted estimates were imprecise and markedly attenuated after accounting for LVEF. Prospective studies integrating ischemia, scar, viability, and longitudinal follow-up are needed.

## 1. Introduction

A coronary chronic total occlusion (CTO) is generally defined as complete epicardial coronary occlusion with Thrombolysis in Myocardial Infarction (TIMI) grade 0 flow and an estimated duration of at least 3 months. CTO is present in approximately one fifth of patients with clinically significant coronary artery disease (CAD) and frequently coexists with prior myocardial infarction (MI) [1,2]. Contemporary consensus documents and guidelines position CTO as a heterogeneous clinical syndrome whose management should integrate symptoms, ischemia, viability, anatomy, procedural risk, and patient preference rather than lesion status alone [3,4,5].

Collateral channels may maintain resting perfusion and viable myocardium, but angiographic collateral grade is an imperfect surrogate for physiological adequacy, inducible ischemia, or hibernation [6,7]. Successful CTO PCI has been associated with reduced ischemic burden in a perfusion study [8]. Regional recovery is most likely when dysfunctional myocardium remains viable and scar burden is limited [9,10]. In a prospective PET/CMR cohort, perfusion improved more consistently than global LV ejection fraction (LVEF) [11]. This heterogeneity may blunt global LVEF changes even when regional mechanical abnormalities are present.

Non-invasive myocardial work combines longitudinal strain with an estimated LV pressure curve and thereby provides a load-informed assessment of total, constructive, and wasted work [12,13,14]. Two-dimensional speckle-tracking global longitudinal strain (GLS) detects systolic dysfunction that may be missed by LVEF and has prognostic value after MI and across cardiovascular disease [15,16,17,18]. Acute coronary ischemia reduces GLS, global work index (GWI), global constructive work (GCW), and global work efficiency (GWE), while global wasted work (GWW) may increase; however, selected observational CTO cohorts have also shown improved GLS after successful revascularization [19,20,21,22]. However, the global and regional myocardial-work phenotype associated with CTO, compared with a diseased multivessel-CAD control group, remains incompletely understood.

We therefore compared LV deformation and myocardial-work indices in stable inpatients with multivessel or left-main CAD with versus without CTO. We examined whether CTO status identified a worse global mechanical phenotype and whether any association attenuated after accounting for prior MI and conventional LV systolic function. Preserved-LVEF and CTO-vessel subgroup analyses were exploratory.

## 2. Materials and Methods

### 2.1. Study Design, Population and Ethics

This retrospective, cross-sectional observational study used an existing clinical-imaging database of 148 unique patients with multivessel or left-main CAD whose echocardiographic examinations were analyzed in the imaging core laboratory using EchoPAC software (EchoPAC PC, v. 112, GE Medical Systems, Milwaukee, WI, USA). Each patient contributed one eligible echocardiographic examination to the analytic cohort.

All included patients were hospitalized at the Department of Cardiovascular Diseases, University Hospital of Split in Split, Croatia from 1 January 2023, until 1 January 2025. All patients were consecutively enrolled and discussed at the institutional Heart Team multidisciplinary meeting involving interventional cardiologists, clinical cardiologists and cardiac surgeons regarding the mode of coronary revascularization. The analysis was performed on a read-only version of the original workbook. Direct identifiers were not exported to analysis results, and all patient data were anonymized and de-identified. The reporting structure followed principles for transparent observational research.

This study was approved by the local Ethics Committee of the University Hospital of Split (filed under approval number 2181-147/01-06/LJ.Z.-25-02, class: 520-03/25-01/153). The research was conducted by adhering to all postulates of good clinical practice and guidelines from the Helsinki declaration (1964, and its later revision in 2013).

### 2.2. Inclusion and Exclusion Criteria

All patients included in the study were 18 years of age or older and have undergone diagnostic coronary angiography within the clinical context of chronic coronary syndrome (CCS) and stable angina. Furthermore, they needed to have a significant multivessel coronary artery disease (MVCAD) that was subsequently confirmed and reviewed by a multidisciplinary Heart Team. In our study, MVCAD was defined angiographically as the presence of significant obstructive coronary artery disease involving at least two major epicardial coronary arteries, with significant stenosis defined as a luminal diameter reduction of ≥70% in the left anterior descending (LAD), left circumflex (LCx), or right coronary artery (RCA), or ≥50% in the left-main (LM) coronary artery. Additional inclusion requirements included the availability of echocardiographic examinations of adequate image quality for the planned analyses and completion of the predefined diagnostic evaluation before study enrolment.

Patients were excluded from the study if they had an acute coronary syndrome (ACS) presentation or if they suffered recent myocardial infarction (within 3 months prior to enrollment) and if they had a presence of concomitant severe valvular heart disease. Additional exclusion criteria comprised severe systemic comorbidities such as severe chronic hepatic dysfunction or liver cirrhosis, severe or end-stage chronic kidney disease, active malignancy and terminal/advanced heart failure.

### 2.3. Adjudication Process, Clinical and Angiographic Variables

For the principal analyses, patients were classified by the source angiographic adjudication as having CTO (n = 70) or no CTO (n = 78). Angiographic adjudication was performed independently by two interventional cardiologists who reviewed the coronary angiograms. Final classification was reached by consensus; in cases of disagreement, a third interventional cardiologist was consulted to resolve discrepancies. CTO was defined angiographically as a coronary lesion with complete interruption of antegrade blood flow (Thrombolysis in Myocardial Infarction [TIMI] flow grade 0) and an estimated duration of occlusion of at least 3 months. The duration of occlusion was determined on the basis of clinical history, prior angiographic documentation, or the timing of a previous acute coronary event in the territory supplied by the occluded vessel.

Recorded variables included age at hospitalization, sex, arterial hypertension, diabetes mellitus, hypercholesterolemia, current smoking, previous MI, previous percutaneous coronary intervention, previous coronary artery bypass grafting, atrial fibrillation, brachial blood pressure, and the presence of significant left-main, left anterior descending (LAD), left circumflex (LCx), and right coronary artery (RCA) disease. Prior revascularization was defined analytically as previous PCI or CABG. Three-vessel disease was derived from the presence of LAD, LCx, and RCA disease. Laboratory variables from blood sampling were captured for each patient and included hemoglobin, creatinine, estimated glomerular filtration rate (eGFR), triglycerides, total cholesterol, HDL cholesterol, LDL cholesterol, fasting glucose, C-reactive protein (CRP), NT-proBNP, and high-sensitivity troponin T (hs-cTnT). Data on chronic medication use were also recorded. Chronic medications were extracted from available data. The source dataset captured cardiac rhythm as sinus rhythm or atrial fibrillation but did not systematically capture QRS duration or intrinsic bundle-branch block classification.

### 2.4. Transthoracic Echocardiography and Myocardial Mechanics

Echocardiographic examination was performed with patients at rest in the standard left lateral decubitus position using a commercially available ultrasound system (Vivid 9, GE Medical Systems, Milwaukee, WI, USA). All data were digitally stored and subsequently analyzed offline using an EchoPAC workstation (version 112, GE Medical Systems), in accordance with the recommendations of the European Association of Cardiovascular Imaging and the American Society of Echocardiography. All echocardiographic measurements and analyses were performed by a single experienced cardiologist trained in speckle-tracking echocardiography (STE).

Left ventricular (LV) volumes and left ventricular ejection fraction (LVEF) were assessed using the biplane Simpson method. Additional measurements included left ventricular end-diastolic volume (LVEDV), left ventricular end-systolic volume (LVESV), left atrial volume (LAV), and the ratio of peak early transmitral inflow velocity to early diastolic mitral annular velocity assessed by tissue Doppler imaging (E/e′). For STE assessment of LV global longitudinal strain (LVGLS), three standard apical views (four-chamber, two-chamber, and apical long-axis) were acquired at a frame rate of 50–60 frames/s. A 17-segment LV model displayed as a bull’s-eye plot was used. After careful delineation of the endocardial border to define the region of interest, the software automatically calculated LVGLS as the mean longitudinal strain across all 17 segments. GLS was analyzed as a continuous magnitude (absolute GLS) without application of a dichotomous normal/abnormal threshold.

Based on the measured LVGLS and brachial systolic blood pressure obtained using a cuff, EchoPAC automatically generated the non-invasive LV pressure–strain loop (PSL), from which myocardial-work (MW) parameters were derived: global work index (GWI), global constructive work (GCW), global wasted work (GWW), and global work efficiency (GWE). The following reference values for myocardial-work parameters were used: GWI ≥ 1290 mmHg%, GCW ≥ 1597 mmHg%, GWE ≥ 90%, and GWW ≤ 238 mmHg%. The analayzed dataset contained raw measurements for 17 LV segments [23]. Coronary dominance and patient-specific perfusion territories were unavailable. Although the export also contained predefined vessel-territory summaries, these were based on standard segment mapping and could not be converted into patient-specific CTO territories without coronary-dominance and perfusion-territory data.

### 2.5. Statistical Analysis

Continuous variables are summarized as mean (SD) and median (IQR); categorical variables as number/denominator (percentage). Baseline balance is expressed with standardized mean differences (SMDs), without significance testing. Crude outcome distributions were compared with the Mann–Whitney U test.

For each global endpoint, the CTO-negative versus CTO-positive mean difference was estimated by ordinary least-squares regression with HC3 heteroscedasticity-consistent covariance and Student t confidence intervals. To ensure that the sequential attenuation analysis reflected covariate adjustment rather than changes in the analyzed population, all four sequential models were re-estimated in a common complete-case cohort of 133 patients with complete global mechanics, LVEF, and the prespecified clinical covariate data. Models were presented sequentially: unadjusted; adjusted for age and sex; the primary clinical model adjusted for age, sex, hypertension, diabetes, and prior MI; and an attenuation model adding LVEF. LVEF was separated because it overlaps with, and may lie downstream of, the myocardial-dysfunction pathway rather than functioning solely as a baseline confounder. For myocardial-work endpoints, an additional sensitivity model included systolic blood pressure because cuff pressure contributes to work estimation. No a priori sample-size calculation was available for this retrospective analysis; all eligible consecutive patients were included, and interpretation emphasized effect-size precision rather than achieved significance.

Holm family-wise adjusted *p*-values across absolute GLS, GWI, GCW, GWW, and GWE were calculated within each model; Benjamini–Hochberg false-discovery-rate values were supportive. Influence, leverage, residual distribution, variance inflation, 1st/99th-percentile winsorization, rank-normalized outcomes, restriction to patients without prior MI, calendar-year adjustment, and exclusion of atrial fibrillation were examined as sensitivity analyses. GWW was additionally log transformed. In an exploratory medication-confounding analysis, patients with complete medication data, global mechanics, and LVEF (n = 88) were modeled with the clinical covariates before and after adding beta-blocker use and renin–angiotensin-system inhibitor use (ACE inhibitor, ARB, or ARNI). Because bundle-branch block data were not systematically available, no conduction-specific sensitivity analysis could be performed.

Within exact single-vessel CTO patients, RCA versus LAD/LCx global mechanics were compared with minimally adjusted models containing age, sex, and prior MI; Holm correction was applied across five global endpoints. Provisional affected-versus-remote segment analyses used Wilcoxon signed-rank tests, with Holm correction across signed longitudinal strain, GWI, GWE, and time to peak, and supportive paired t estimates. These regional analyses were regarded as mapping-sensitivity audits because patient-specific perfusion territories were unavailable. Missing outcomes were not imputed. Statistical analyses were performed by using R software (version 4.5.3; http://www.r-project.org). Nominal two-sided *p*-values and 95% CIs are reported.

## 3. Results

### 3.1. Cohort and Clinical Characteristics

**Figure 1:**
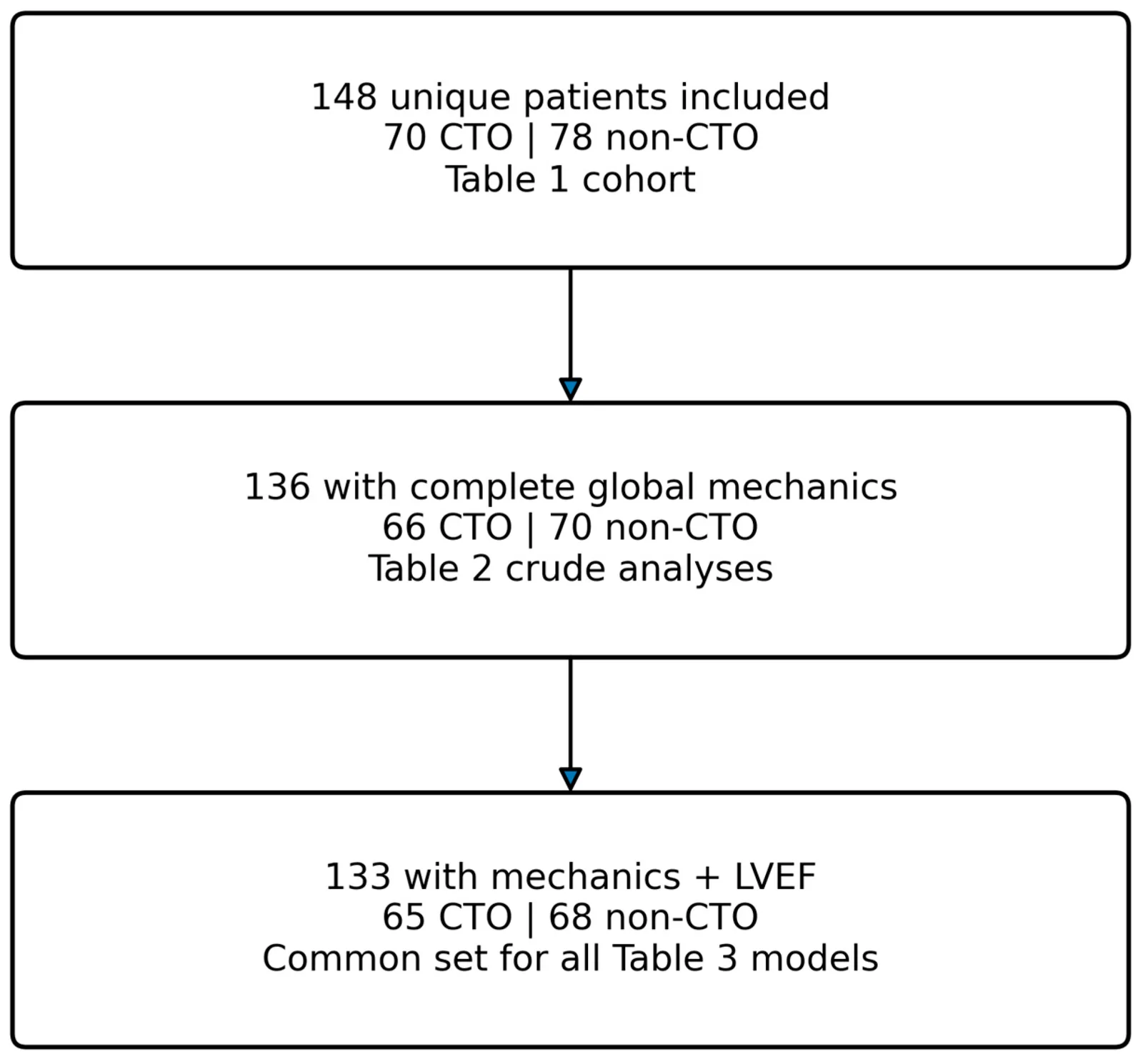
Participant flow and analysis sets. The full cohort comprised 148 unique patients. Crude global mechanics analyses used the 136 patients with complete global mechanics, while all sequential HC3 regression models used the same common complete-case cohort of 133 patients with complete global mechanics and LVEF data. CTO indicates chronic total occlusion; LVEF, left ventricular ejection fraction.

The analyzed cohort included 148 unique patients: 70 with CTO and 78 without CTO. Core global mechanics were complete in 136 patients: 66 (94.3%) with CTO and 70 (89.7%) without CTO. LVEF information was available in 142 patients, and 133 patients had complete global mechanics and LVEF data pairs; this common complete-case cohort comprised 65 patients with CTO and 68 without CTO and was used for all sequential regression models (Figure 1). Clinical covariates in the primary model were fully complete.

The median age was 67.0 years (IQR, 60.0–74.8) in the CTO group and 68.5 years (IQR, 62.0–73.0) in the non-CTO group. Women comprised 15.7% and 28.2%, respectively (SMD, −0.31). Prior MI was more frequent with CTO (30/70 [42.9%] vs. 18/78 [23.1%]; SMD, 0.43). Systolic blood pressure was closely balanced (mean, 137.7 vs. 137.2 mmHg; SMD, 0.02). Patients with CTO had lower LVEF (median, 47.0% [IQR, 41.8–53.0%] vs. 51.0% [IQR, 44.0–59.0%]; SMD, −0.33), larger LV end-diastolic volume (124.0 [IQR, 100.0–141.8] vs. 105.0 [IQR, 88.2–123.0] mL; SMD, 0.54), and larger LV end-systolic volume (62.0 [IQR, 47.0–78.8] vs. 50.0 [IQR, 35.8–64.5] mL; SMD, 0.56) (Table 1).

Baseline laboratory variables are now reported together with chronic medication use in Table 1. Among variables with available data, triglycerides showed the largest standardized imbalance (median, 1.7 vs. 1.4 mmol/L; SMD, 0.59), whereas hemoglobin, renal function, CRP, NT-proBNP, and hs-cTnT showed smaller standardized differences. Chronic medication data were available in 100 patients (40 with CTO and 60 without CTO); beta-blocker use was 45.0% in both groups, while ACE-inhibitor use was 37.5% versus 53.3%, respectively.

Among the 10 patients with CTO and a history of prior PCI, the CTO involved the RCA in 4 patients, LAD in 3, and LCx in 3; 9 of these 10 patients also had a prior MI. The vessel and segment treated during the previous PCI were not retained in the source dataset, so same-vessel or same-segment concordance between prior PCI and the subsequently identified CTO could not be established.

### 3.2. Global Longitudinal Strain and Myocardial Work

**Figure 2:**
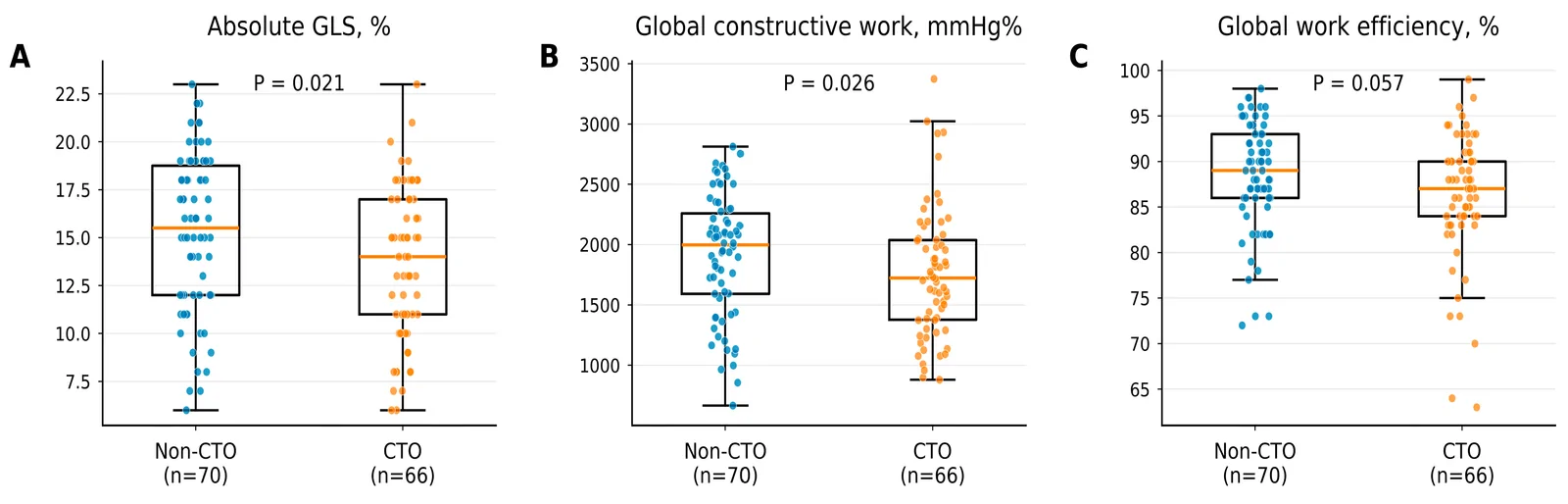
Crude distributions of global LV mechanics by CTO status. Individual observations are overlaid on box plots for absolute global longitudinal strain (**A**), global constructive work (**B**), and global work efficiency (**C**). Center lines are medians, boxes are interquartile ranges, and whiskers extend to the most extreme observation within 1.5 interquartile ranges. *p*-values are from two-sided Mann–Whitney U tests and therefore test distributions/ranks rather than the HC3 regression mean difference. CTO indicates chronic total occlusion; GLS, global longitudinal strain.

Median absolute GLS was lower among patients with CTO (14.0% [IQR, 11.0–17.0%]) than without CTO (15.5% [IQR, 12.0–18.8%]; Mann–Whitney *p* = 0.021). Median GCW was also lower (1723 [IQR, 1377–2038] vs. 1997 [IQR, 1592–2260] mmHg%; *p* = 0.026), while GWE was directionally lower, and this result was of borderline statistical significance (87.0% [IQR, 84.0–90.0%] vs. 89.0% [IQR, 86.0–93.0%]; *p* = 0.057). The GWI and GWW distributions did not clearly differ between the two groups (Table 2 and Figure 2).

In the common complete-case cohort (n = 133), HC3 regression yielded an unadjusted CTO− vs. CTO+ absolute GLS difference of −1.40 percentage points (95% CI, −2.80 to 0.00; *p* = 0.051; Hedges g, −0.34). The corresponding unadjusted differences were −95.4 mmHg% (95% CI, −257.2 to 66.4) for GWI, −136.1 mmHg% (95% CI, −316.3 to 44.0) for GCW, 29.6 mmHg% (95% CI, −18.3 to 77.5) for GWW, and −2.40 percentage points (95% CI, −4.67 to −0.14) for GWE. The distinction between the rank-based distributional tests using all 136 patients with global mechanics and the common-cohort HC3 mean-difference estimates is shown in Table 2 and Table 3.

**Figure 3:**
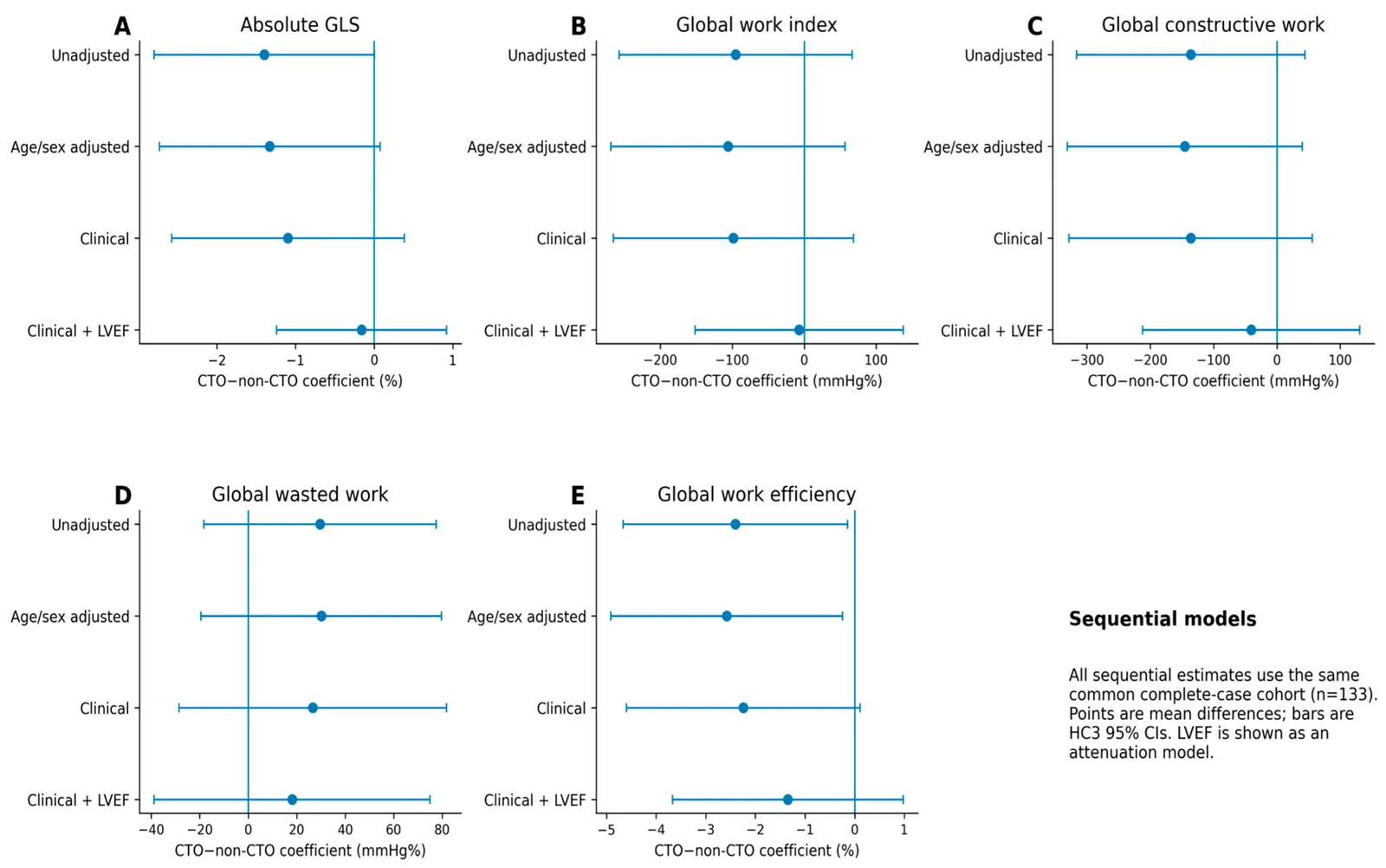
CTO coefficients across sequential models for global LV mechanics. Points are CTO− vs. CTO+ coefficients and horizontal bars are 95% CIs from ordinary least-squares models with HC3 robust covariance. All sequential estimates are derived from the same common complete-case cohort of 133 patients. Models are unadjusted; adjusted for age and sex; clinically adjusted for age, sex, hypertension, diabetes, and prior MI; and the clinical model plus LVEF as an attenuation analysis. A negative coefficient indicates a lower outcome with CTO, except that a positive GWW coefficient indicates more wasted work. GLS, global longitudinal strain; LVEF, LV ejection fraction; MI, myocardial infarction.

After primary clinical adjustment in the same 133-patient cohort, the CTO coefficient was −1.10 percentage points (95% CI, −2.58 to 0.38; *p* = 0.145) for absolute GLS; −98.3 mmHg% (95% CI, −264.9 to 68.2) for GWI; −136.2 mmHg% (95% CI, −328.3 to 55.9) for GCW; 26.6 mmHg% (95% CI, −28.5 to 81.8) for GWW; and −2.24 percentage points (95% CI, −4.60 to 0.11) for GWE. None of these five clinical-model coefficients remained below the 0.05 statistical significance threshold after Holm correction. Adding LVEF to the model markedly attenuated the absolute GLS, GWI, and GCW coefficients (absolute GLS, −0.16 percentage points [95% CI, −1.24 to 0.92]; GWI, −7.1 mmHg% [95% CI, −151.6 to 137.3]; GCW, −40.7 mmHg% [95% CI, −212.2 to 130.8]) (Table 3 and Figure 3).

### 3.3. Exploratory CTO-Territory Analyses

Among patients with CTO, RCA CTO showed nominally more favorable global mechanics than LAD/LCx CTO, but no endpoint survived Holm correction across the five global measures (Supplementary Table S1 and Supplementary Figure S1). No additional angiographic fields in the source dataset permitted authoritative patient-specific assignment of LV segments to the CTO perfusion territory. Results under three plausible mappings are therefore provided only as a provisional reproducibility audit (Supplementary Table S2) and should not be interpreted as evidence of territory-specific dysfunction.

### 3.4. Sensitivity and Exploratory Analyses

Primary-model variance inflation factors were low (maximum, 1.26). Estimates were directionally similar after winsorization, rank normalization, exclusion of atrial fibrillation, and systolic-pressure adjustment for myocardial-work endpoints. Deleting observations exceeding Cook D greater than 4/n strengthened some estimates, indicating nontrivial influence but no directional reversal. The right-skewed GWW result remained null after log transformation. In an exploratory medication-confounding analysis restricted to 88 patients with complete medication data, global mechanics, and LVEF, adding beta-blocker and renin–angiotensin-system inhibitor use to the clinical model produced little change in the CTO coefficient for absolute GLS (−0.75 to −0.69 percentage points) or GWE (−2.67 to −2.71 percentage points); confidence intervals continued to include no difference.

In a preserved-LVEF exploratory analysis, 69 patients had LVEF of 50% or greater and available GLS (27 with CTO and 42 without CTO). Mean absolute GLS was 16.3% in the CTO group and 17.3% in the non-CTO group. The CTO− versus CTO+ difference was −1.05 percentage points (95% CI, −2.59 to 0.49; *p* = 0.179) unadjusted and −0.84 percentage points (95% CI, −2.28 to 0.61; *p* = 0.251) after adjustment for age, sex, hypertension, diabetes, and prior MI. GLS was therefore retained as a continuous measure rather than dichotomized using a post hoc threshold.

## 4. Discussion

In this multivessel/left-main CAD cohort, CTO status was associated with a modest adverse global LV mechanical phenotype. Absolute GLS and GCW were lower in rank-based crude comparisons, and GWE was directionally lower. In the common complete-case regression cohort, the unadjusted absolute GLS estimate was small-to-moderate and borderline in precision, while clinical adjustment make this differences smaller in magnitude. Adding LVEF as a global parameter of systolic function largely attenuated the global GLS, GWI, and GCW coefficients.

The direction of the global results we observe is biologically plausible and coherent. Acute and transient coronary-occlusion studies show reduced GLS, GWI, GCW, and GWE during ischemia, with reversal after reperfusion [19,20,21]. In chronic cardiac disease, lower GCW may reflect less effective systolic shortening under the prevailing pressure load, while a weak GWW signal may occur when scar or reduced force generation predominates over paradoxical lengthening. The absence of a clear GWI or GWW difference therefore does not negate an adverse deformation phenotype, but it emphasizes the modest magnitude and heterogeneity of the global association.

Prior MI was almost twice as common in the CTO group and LVEF was lower, with larger ventricular volumes. The attenuation sequence is therefore informative—much of the global mechanical contrast was shared with established myocardial injury and remodeling. Prior MI may be both a common cause and part of the causal history of CTO, whereas LVEF is another expression of myocardial dysfunction and may lie downstream of chronic ischemia and infarction. Conditioning on LVEF addresses whether CTO contains incremental mechanical information among patients with similar conventional systolic function—it should not be perceived as the only valid causal estimand.

Sex distribution also differed between groups: women represented 28.2% of the non-CTO group but only 15.7% of the CTO group. Sex-related differences have been described in coronary plaque burden, composition, and vascular remodeling, and normal reference values for both GLS and myocardial-work indices also vary by sex [14,24,25]. Although sex was included in all adjusted models, only 33 women were enrolled overall, including 11 with CTO. This limited sample precluded stable sex-stratified or interaction analyses, and residual sex-related confounding cannot be excluded.

Chronic medical therapy may also be associated with both disease severity and myocardial mechanics and therefore represents a potential source of residual confounding. In the subset with complete medication data, additional adjustment for beta-blocker and renin–angiotensin-system inhibitor use did not significantly alter the main CTO coefficients, with a notion that medication use data were incomplete and the cross-sectional design is vulnerable to confounding by indication. These sensitivity findings should therefore be considered reassuring but not definitive.

The segment audit illustrates a broader and important limitation of regional mechanics derived from clinical exports. CTO-related ischemia is territorial, and a global GLS value can dilute a localized mechanical abnormality, particularly in patients with multivessel disease, prior MI, or global LV remodeling. Although segment-level strain and work data were available, patient-specific coronary dominance, quantitative perfusion territories, and sufficiently granular angiographic information for reliable segment-to-CTO assignment were not available. Angiographic collateral appearance also correlates imperfectly with collateral physiology, ischemia, and viability [6,7], and regional recovery after recanalization is greatest when scar burden is limited [9,10]. Accordingly, the inability to establish a robust spatial relationship between CTO location and regional mechanical impairment should be viewed as a limitation of our study, and the affected-versus-remote estimates were not used to support the main conclusion.

The preserved-LVEF analysis clearly reiterates why deformation imaging should be viewed as clinically relevant even when conventional systolic function is preserved. In this subgroup, absolute GLS was numerically lower with CTO, but the continuous between-group difference was imprecise and compatible with little or no difference after clinical adjustment. Moreover, LV GLS can provide prognostic information beyond LVEF [17,18], but reference values vary by vendor, loading conditions, age, sex, and imaging laboratory [15,16,25]. These data therefore do not support a universal dichotomous GLS threshold in this cohort.

These findings do not imply benefit from CTO PCI. EuroCTO found improved angina and health status with CTO PCI in selected patients, whereas longer-term hard-event differences remained uncertain [26,27]. DECISION-CTO did not show a clinical-outcome advantage and was limited by early termination and crossover [28]. REVASC and EXPLORE did not demonstrate routine improvement in their primary global or regional LV-function endpoints [29,30], while OPEN-CTO documented health-status gains in a prospective observational registry [31]. Current revascularization guidance accordingly places the clearest emphasis on symptom relief and individualized selection rather than the presumed recovery of global LV function [3,4,5,32]. A longitudinal study combining strain/work with ischemia and viability imaging would be required to determine whether the abnormalities observed here are reversible.

This study has several strengths. The comparator group had advanced CAD without CTO rather than healthy controls, the analysis reported effect sizes with robust CIs, the retrospectively assigned endpoint hierarchy was made explicit, and a source-formula audit prevented unreliable regional summaries from being promoted.

There are some important limitations associated with our work. This was a single-center, retrospective cross-sectional analysis with a limited patient sample, and possible referral and selection bias. Residual confounding is likely, and calendar year differed between groups. Furthermore, the database did not include information on quantitative ischemia, scar presence and its extent, viability, coronary dominance, collateral physiology, CTO duration, procedural complexity, or longitudinal clinical outcomes. The vessel and segment of previous PCI were not retained, preventing distinction between a CTO involving a previously treated coronary territory and a CTO arising in a different vessel. Patient-specific CTO perfusion territories could not be assigned reliably, which limits mechanistic interpretation of global strain differences. The source dataset captured rhythm but not QRS duration or intrinsic bundle-branch block classification; therefore, unmeasured LBBB or other intraventricular conduction delay could have affected GLS and myocardial-work indices, particularly GWW and GWE [33]. Medication data were incomplete, despite a supportive subset sensitivity analysis. Global myocardial work depends on cuff pressure and standardized pressure-curve assumptions. Normal strain varies by segment, and remote territories may also be abnormal in multivessel CAD. Finally, no treatment comparison was present, precluding inference about the effect of revascularization.

## 5. Conclusions

Among inpatients with multivessel and/or left-main coronary artery disease, the presence of a CTO was associated with a modestly adverse global LV mechanical profile, most notably reflected by lower crude absolute GLS and GCW. However, these associations became less precise after adjustment for clinical covariates and were further attenuated after additional adjustment for LVEF. This pattern suggests that the observed differences in global myocardial mechanics are, at least in part, explained by the close interrelationship between CTO status, prior myocardial infarction, conventional LV systolic dysfunction, and adverse ventricular remodeling. Future studies in larger, prospectively characterized cohorts are needed to determine whether CTO is independently associated with impaired myocardial mechanics beyond established measures of LV function and scar burden, and to clarify whether advanced deformation and myocardial-work indices provide incremental prognostic or therapeutic information in this population.

## Figures and Tables

**Table 1:** Clinical, angiographic, echocardiographic, laboratory, and chronic medication characteristics by CTO status. Data are mean (SD), median [IQR], or n/N (%). Available n is shown for laboratory variables with missing data, and medication rows display the available group-specific denominator. SMD is calculated as CTO minus non-CTO; absolute values of 0.10, 0.20, and 0.50 are commonly interpreted as small, moderate, and large imbalance descriptors, not hypothesis tests.

Characteristic	Non-CTO (n = 78)	CTO (n = 70)	SMD
Age, years	67.7 (7.8)	66.6 (10.2)	−0.12
Female sex	22 (28.2%)	11 (15.7%)	−0.31
Arterial hypertension	59 (75.6%)	48 (68.6%)	−0.16
Diabetes mellitus	37 (47.4%)	27 (38.6%)	−0.18
Hypercholesterolemia	31 (39.7%)	17 (24.3%)	−0.34
Current smoking	29 (37.2%)	25 (35.7%)	−0.03
Prior myocardial infarction	18 (23.1%)	30 (42.9%)	0.43
Prior PCI	5 (6.4%)	10 (14.3%)	0.26
Prior CABG	3 (3.8%)	4 (5.7%)	0.09
Atrial fibrillation	1 (1.3%)	2 (2.9%)	0.11
Heart rate, beats/min	77.3 (14.3)	77.4 (15.9)	0.01
Systolic blood pressure, mmHg	137.2 (19.5)	137.7 (22.8)	0.02
Diastolic blood pressure, mmHg	77.8 (11.2)	78.9 (13.8)	0.09
Significant left-main (LM) stenosis	18/78 (23.1%)	17/70 (24.3%)	0.03
Significant LAD stenosis	67/78 (85.9%)	62/70 (88.6%)	0.08
Significant circumflex (Cx) stenosis	68/78 (87.2%)	60/70 (85.7%)	−0.04
Significant RCA stenosis	70/78 (89.7%)	67/70 (95.7%)	0.23
Left ventricular ejection fraction (LVEF), %	51.0 [44.0, 59.0]	47.0 [41.8, 53.0]	−0.33
LV end-diastolic volume (LVEDV), mL	105.0 [88.2, 123.0]	124.0 [100.0, 141.8]	0.54
LV end-systolic volume (LVESV), mL	50.0 [35.8, 64.5]	62.0 [47.0, 78.8]	0.56
E/e′ ratio	11.0 [8.9, 16.8]	10.2 [8.6, 13.0]	−0.12
**Laboratory variables**			
Hemoglobin, g/L	142 [129, 151]	140 [130, 153]	−0.02
Creatinine, micromol/L	86 [79, 104]	92 [80, 105]	0.00
eGFR, mL/min/1.73 m^2^	69.8 [54.1, 87.0]	69.1 [53.8, 82.5]	0.04
Triglycerides, mmol/L	1.4 [1.2, 1.9]	1.7 [1.2, 2.7]	0.59
Total cholesterol, mmol/L	4.6 [3.7, 5.3]	4.4 [3.7, 5.8]	0.29
HDL cholesterol, mmol/L	1.1 [0.9, 1.2]	1.0 [0.8, 1.2]	−0.04
LDL cholesterol, mmol/L	2.6 [1.9, 3.5]	2.5 [2.1, 3.7]	0.24
Fasting glucose, mmol/L	7.6 [6.0, 10.4]	7.7 [6.3, 9.7]	−0.03
CRP, mg/L	5.5 [1.5, 30.9]	5.3 [1.8, 24.7]	−0.17
NT-proBNP, pg/mL	1218 [222, 3649]	1810 [472, 4024]	−0.15
High-sensitivity troponin T, ng/L	60 [15, 248]	58 [24, 565]	0.12
**Chronic medications**			
Aspirin	25/60 (41.7%)	14/40 (35.0%)	−0.14
P_2_Y_12_ inhibitor	4/60 (6.7%)	4/40 (10.0%)	0.12
Beta-blocker	27/60 (45.0%)	18/40 (45.0%)	0.00
ACE inhibitor	32/60 (53.3%)	15/40 (37.5%)	−0.32
Angiotensin receptor blocker	4/60 (6.7%)	3/40 (7.5%)	0.03
ARNI	0/60 (0.0%)	2/40 (5.0%)	0.32
High-intensity statin	31/60 (51.7%)	17/40 (42.5%)	−0.18
Direct oral anticoagulant	3/60 (5.0%)	5/40 (12.5%)	0.27
Warfarin	4/60 (6.7%)	0/40 (0.0%)	−0.38
Diuretic	24/60 (40.0%)	10/40 (25.0%)	−0.32
Antidiabetic medication	25/60 (41.7%)	14/40 (35.0%)	−0.14
Calcium-channel blocker	26/60 (43.3%)	13/40 (32.5%)	−0.22

**Table 2:** Crude global longitudinal strain and myocardial-work measures by CTO status. Group data are median [IQR]. *p*-values are from two-sided Mann–Whitney U tests. Absolute GLS is reported as magnitude; lower values indicate worse deformation. Work-index units are mmHg%.

Outcome	Non-CTO, Median [IQR]	CTO, Median [IQR]	*p*-Value
Absolute global longitudinal strain (GLS), %	15.5 [12.0, 18.8]	14.0 [11.0, 17.0]	0.021
Global work index (GWI), mmHg%	1554 [1159, 1835]	1398 [1166, 1632]	0.109
Global constructive work (GCW), mmHg%	1997 [1592, 2260]	1723 [1377, 2038]	0.026
Global wasted work (GWW), mmHg%	190 [116, 278]	191 [140, 296]	0.487
Global work efficiency (GWE), %	89.0 [86.0, 93.0]	87.0 [84.0, 90.0]	0.057

**Table 3:** CTO− vs. CTO+ coefficients across sequential HC3 regression models in the common complete-case cohort. All models use the same 133 patients (65 CTO and 68 non-CTO). Values are coefficient (95% CI); *p*-value. The clinical model includes age, sex, hypertension, diabetes, and prior MI. The LVEF model is an attenuation/sensitivity analysis because LVEF may share or mediate the myocardial-dysfunction pathway. Holm adjustment was applied across the five outcomes within each model.

Outcome	Model	CTO− vs. CTO+ (95% CI)	*p*	Holm *p*
Absolute GLS, percentage points	Unadjusted	−1.40 (−2.80 to 0.00)	0.051	0.203
	Age/sex adjusted	−1.33 (−2.73 to 0.08)	0.063	0.253
	Clinical	−1.10 (−2.58 to 0.38)	0.145	0.581
	Clinical + LVEF	−0.16 (−1.24 to 0.92)	0.768	1.000
GWI, mmHg%	Unadjusted	−95.4 (−257.2 to 66.4)	0.245	0.447
	Age/sex adjusted	−105.9 (−268.7 to 56.8)	0.200	0.400
	Clinical	−98.3 (−264.9 to 68.2)	0.245	0.581
	Clinical + LVEF	−7.1 (−151.6 to 137.3)	0.922	1.000
GCW, mmHg%	Unadjusted	−136.1 (−316.3 to 44.0)	0.137	0.412
	Age/sex adjusted	−145.3 (−330.8 to 40.3)	0.124	0.372
	Clinical	−136.2 (−328.3 to 55.9)	0.163	0.581
	Clinical + LVEF	−40.7 (−212.2 to 130.8)	0.639	1.000
GWW, mmHg%	Unadjusted	29.6 (−18.3 to 77.5)	0.223	0.447
	Age/sex adjusted	30.2 (−19.5 to 79.8)	0.231	0.400
	Clinical	26.6 (−28.5 to 81.8)	0.341	0.581
	Clinical + LVEF	18.1 (−38.8 to 75.0)	0.530	1.000
GWE, percentage points	Unadjusted	−2.40 (−4.67 to −0.14)	0.037	0.187
	Age/sex adjusted	−2.58 (−4.91 to −0.25)	0.031	0.153
	Clinical	−2.24 (−4.60 to 0.11)	0.061	0.306
	Clinical + LVEF	−1.35 (−3.67 to 0.97)	0.253	1.000

## Data Availability

The data supporting the findings of this study are available from the corresponding author upon reasonable request. The datasets are not publicly available due to privacy, ethical, and institutional restrictions related to the protection of patient-level clinical data. Any data sharing will be subject to applicable institutional approval and data-protection regulations.

## Supplementary Materials

The following supporting information can be downloaded at: https://www.mdpi.com/article/10.3390/jcdd13090449/s1, Table S1: Exploratory global mechanics in RCA versus LAD/LCx CTO; Table S2: Provisional affected-versus-remote estimates under three segment maps; Figure S1: Global LV mechanics in RCA versus LAD/LCx CTO; Figure S2: Mapping sensitivity of affected-versus-remote estimates.
