# Supplementary material for: Left Ventricular Mechanics in Multivessel Coronary Artery Disease with Versus Without Chronic Total Occlusion
Source: J Cardiovasc Dev Dis. 2026 Sep 9;13(9):449. doi: 10.3390/jcdd13090449 (PMC13607136; doi:10.3390/jcdd13090449)
Supplement: Supplementary file 1 [file jcdd-13-00449-s001.zip › jcdd-4534395-supplementary.pdf]

Supplementary Material

Supplementary Table S1. Exploratory global mechanics in RCA versus LAD/LCx CTO.Models were adjusted for age, sex, and prior MI. Holm correction was applied across five global mechanics endpoints.

Outcome	Model	RCA minus LAD/LCx (95% CI)	P	Holm P
Absolute GLS, percentage points	Unadjusted	2.20 (0.14 to 4.26)	0.036	0.145
	Age/sex/prior MI	2.34 (0.29 to 4.39)	0.026	0.129
GWI, mmHg%	Unadjusted	256.9 (34.7 to 479.2)	0.024	0.121
	Age/sex/prior MI	257.8 (30.3 to 485.3)	0.027	0.129
GCW, mmHg%	Unadjusted	283.2 (13.3 to 553.2)	0.040	0.145
	Age/sex/prior MI	279.8 (-1.4 to 561.0)	0.051	0.153
GWW, mmHg%	Unadjusted	-60.3 (-158.1 to 37.4)	0.222	0.222
	Age/sex/prior MI	-60.5 (-161.4 to 40.5)	0.236	0.236
GWE, percentage points	Unadjusted	4.17 (-0.01 to 8.35)	0.051	0.145
	Age/sex/prior MI	4.13 (-0.09 to 8.36)	0.055	0.153

Supplementary Table S2. Provisional affected-versus-remote estimates under three segment maps. Literal AHA numeric suffixes, label-normalized anatomy, and source-formula assignments are compared as a reproducibility audit.

**Measure**	**Segment map**	**Mean paired difference (95% CI)**	**Wilcoxon P**	**Holm P**
Signed longitudinal strain, percentage points	Label-normalized anatomy	1.21 (0.08 to 2.34)	0.032	0.097
Signed longitudinal strain, percentage points	Canonical numeric suffix	1.84 (0.73 to 2.94)	<.001	0.003
Signed longitudinal strain, percentage points	Source-formula assignment	1.43 (0.38 to 2.47)	0.006	0.024
GWI, mmHg%	Label-normalized anatomy	-142.5 (-227.2 to -57.9)	0.003	.010
GWI, mmHg%	Canonical numeric suffix	-97.8 (-182.6 to -12.9)	0.022	0.067
GWI, mmHg%	Source-formula assignment	-80.5 (-164.1 to 3.1)	0.062	0.185

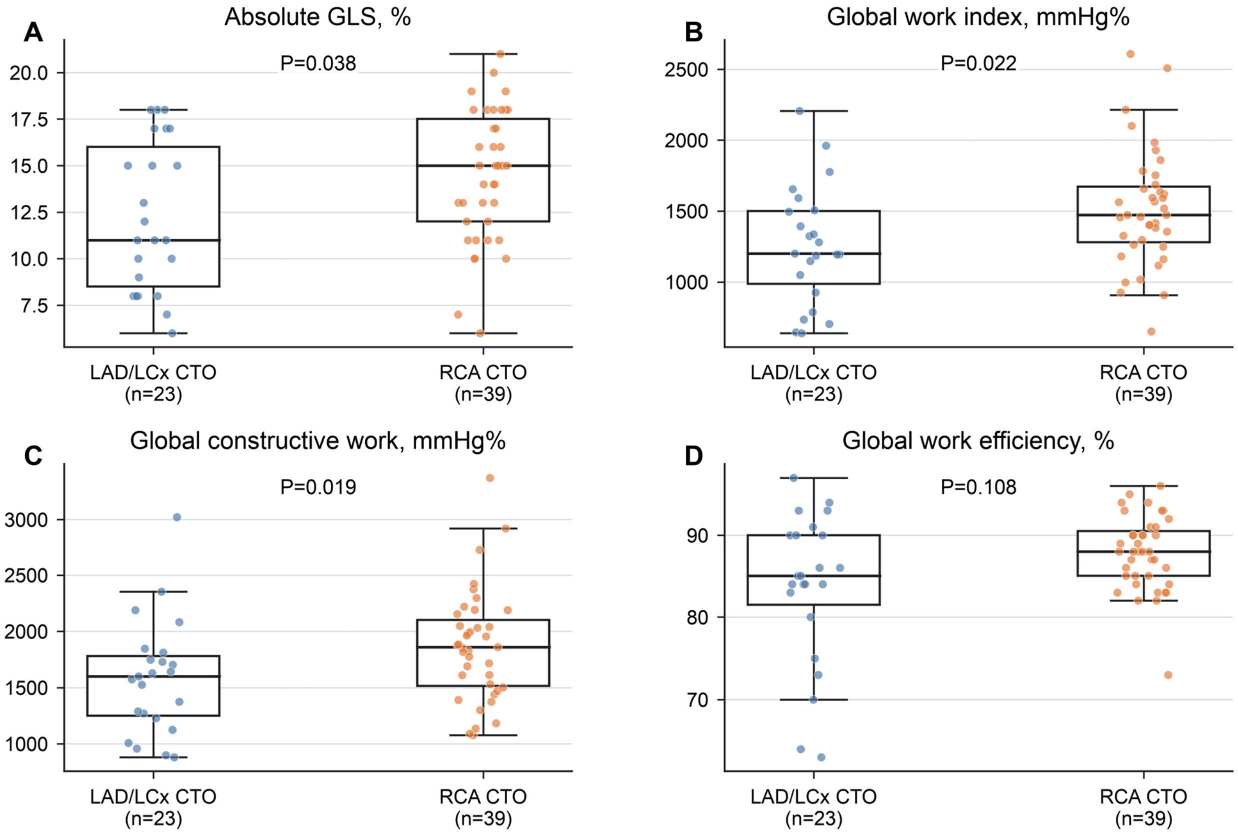

Supplementary Figure S1. Global LV mechanics in RCA versus LAD/LCx CTO. Individual observations and distribution summaries are shown for exact single-territory CTO codes. Mixed-territory codes were excluded. P values are exploratory and were not below .05 after Holm correction across the five global mechanics endpoints.

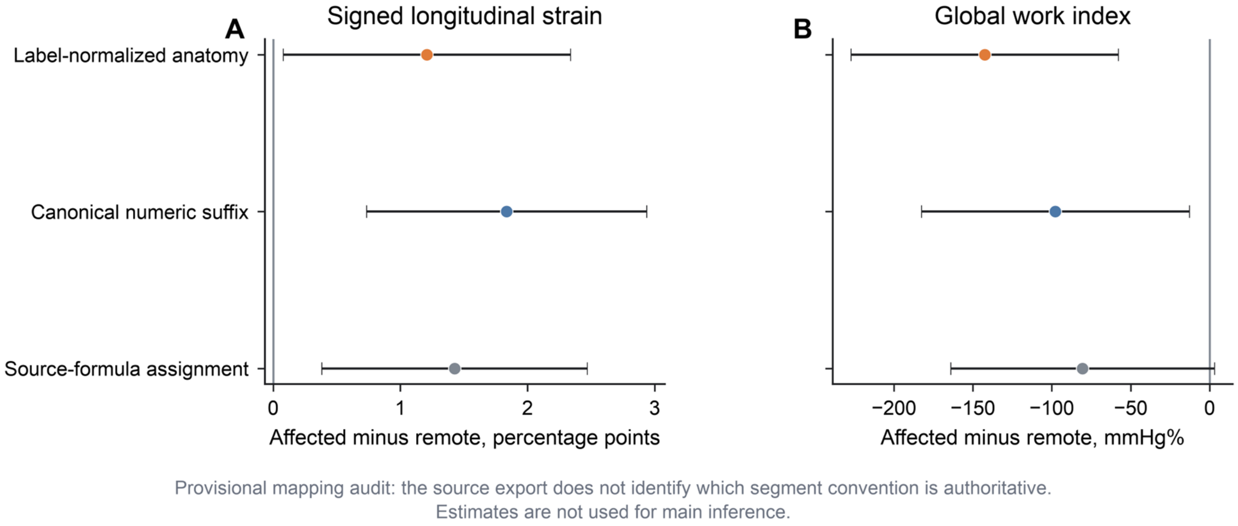

Supplementary Figure S2. Mapping sensitivity of affected-versus-remote estimates. Mean paired differences and 95% CIs for signed longitudinal strain and global work index are shown under label-normalized anatomical, canonical numeric-suffix, and source-formula assignments. The source export does not establish which convention is authoritative; estimates are provided as a reproducibility audit and are not used for main inference.
